# Clinical Characteristics and Outcomes of Anti-MDA5 Antibody-Associated Interstitial Lung Disease: A Single-Center Retrospective Cohort Study from an Integrated Advanced Lung Disease and Lung Transplant Program in the US

**DOI:** 10.3390/jcm15197623

**Published:** 2026-10-01

**Authors:** Shambhu Aryal, Shourjo Chakravorty, Ho Cheol Kim, Ashima Malik, Pragya Singh, Ramona Raya, Oksana A. Shlobin, Christopher S. King, Steven D. Nathan, Zein Kattih

**Affiliations:** 1 Advanced Lung Disease and Transplant Program, Inova Schar Heart and Vascular Institute, Inova Health System, 3300 Gallows Rd, Falls Church, VA 22042, USA; 2 Division of Pulmonary and Critical Care Medicine, Thomas Jefferson Hospital, 111 S 11th S, Philadelphia, PA 19107, USA; 3 Department of Pulmonary & Critical Care Medicine, Asan Medical Center, University of Ulsan College of Medicine, 88, Olympic-ro 43-gil, Songpa-gu, Seoul 05505, Republic of Korea; 4 Division of Rheumatology, Inova Fairfax Hospital, 3300 Gallows Rd, Falls Church, VA 22042, USA

**Keywords:** anti-MDA5, dermatomyositis, interstitial lung disease, rapidly progressive interstitial lung disease, lung transplantation, pulmonary hypertension, tofacitinib, myositis

## Abstract

**Background/Objectives:** Anti-melanoma differentiation-associated gene 5 (anti-MDA5) antibody is associated with dermatomyositis/clinically amyopathic dermatomyositis and a high risk of rapidly progressive interstitial lung disease (RP-ILD). **Methods:** We retrospectively analyzed 34 consecutive anti-MDA5-positive patients evaluated at a tertiary Advanced Lung Disease and Lung Transplant center between 1 January 2018 and 31 December 2024. **Results:** Of 1720 interstitial lung disease (ILD) evaluations, 34 (2.0%) were anti-MDA5 positive (median age 51 years; 56% female). ILD was present in 85%; RP-ILD developed in 13 patients (38%). Overall mortality was 32%, with 11 of 13 deaths occurring in the RP-ILD subgroup (85% mortality). Six patients were listed for lung transplantation and three underwent transplantation; all three remained alive at last follow-up (24–49 months). Right-heart catheterization performed in 11 patients demonstrated pulmonary hypertension (PH) in the majority (64%). Inpatient presentation and elevated ferritin were associated with RP-ILD; elevated CRP and RP-ILD predicted mortality. **Conclusions:** We conclude that anti-MDA-5-associated ILD carries high mortality when RP-ILD develops. Early recognition, aggressive immunosuppression, systematic consideration of PH, and timely referral for lung transplantation can improve outcomes in selected patients. Routine screening for anti-MDA-5 antibodies should be considered in younger patients with new or progressive ILD.

## 1. Introduction

Anti-melanoma differentiation-associated gene 5 (anti-MDA5) antibody is a relatively uncommon myositis-specific autoantibody linked to dermatomyositis (DM) and clinically amyopathic dermatomyositis (CADM) [1,2]. It carries a well-recognized association with interstitial lung disease (ILD), particularly rapidly progressive ILD (RP-ILD), which is associated with high short-term mortality [3,4,5]. Phenotypic heterogeneity has been described, ranging from severe lung-dominant disease to predominantly cutaneous or vasculopathic presentations [6].

Definitions of RP-ILD vary across studies, commonly encompassing respiratory symptom onset or progression over days to weeks (sometimes up to 3–6 months), often accompanied by radiographic or physiological decline [7]. Management remains challenging and typically involves intensive immunosuppression, including corticosteroids, calcineurin inhibitors, cyclophosphamide, rituximab, intravenous immunoglobulin (IVIG), plasma exchange, and more recently Janus kinase (JAK) inhibitors such as tofacitinib [8,9,10].

Lung transplantation has been reported in selected cases with acceptable short-term outcomes and without recurrence of ILD in the allograft, but published experience remains limited [11]. Comorbid pulmonary hypertension (PH) in anti-MDA5 ILD has received little attention. Data from centers with integrated Advanced Lung Disease (ALD) and Lung Transplant (Ltx) programs are particularly scarce.

We therefore describe the clinical characteristics, treatments, and outcomes of a consecutive cohort of anti-MDA5-positive patients evaluated at a high-volume lung transplant center, with particular attention to RP-ILD, PH, and transplant candidacy.

## 2. Materials and Methods

### 2.1. Study Design and Population

This was a single-center retrospective cohort study. All consecutive patients evaluated by the Inova Advanced Lung Disease and Transplant Program, Virginia (VA), United States of America (USA) between 1 January 2018 and 31 December 2024 who tested positive for anti-MDA5 antibody were included. Patients were eligible regardless of the presence or absence of clinically confirmed ILD, in order to characterize the full pulmonary spectrum of anti-MDA5 positivity. Exclusion criteria were (i) anti-MDA5 testing that was reported as indeterminate and not subsequently confirmed as positive, and (ii) incomplete medical records that precluded determination of vital status. No patients meeting the inclusion criteria were excluded for incomplete vital-status follow-up. The study was approved by the Institutional Review Board (IRB U21-02-4389). The Strengthening of Reporting of Observational Studies in Epidemiology (STROBE) checklist was followed. Anti-MDA5 antibodies were detected by the commercially available myositis-specific autoantibody immunoassay used by the institutional clinical laboratory during the study period. Positivity was defined according to the laboratory-reported reference cutoff in force at the time of testing. The same commercial assay family was used throughout 2018–2024, although reagent lots and numeric cutoff units may have varied over time. Individual optical-density or arbitrary-unit titers were not uniformly archived and were therefore not analyzed as continuous variables.

### 2.2. Clinical Variables and Definitions

Data extracted included demographics, clinical features, serologies, inflammatory markers, imaging, pulmonary function tests (PFTs), right-heart catheterization (RHC) results (when performed), treatments, transplant listing and outcomes, and vital status.

RP-ILD was defined as rapid onset or progression of respiratory symptoms over a period of up to six weeks (or acute-on-chronic deterioration) without an alternative explanation (infection, heart failure, etc.) and with clinical progression despite therapy. Pulmonary hypertension was defined according to the 7th World Symposium on Pulmonary Hypertension/2022 ESC/ERS hemodynamic criteria as a mean pulmonary artery pressure (mPAP) > 20 mmHg on right-heart catheterization. Precapillary PH was defined as mPAP > 20 mmHg, pulmonary capillary wedge pressure (PCWP) ≤ 15 mmHg, and pulmonary vascular resistance (PVR) ≥ 2 Wood units [12].

### 2.3. Statistical Analysis

Descriptive statistics are presented as means/medians with interquartile ranges (IQRs) or counts and percentages. Continuous variables were compared using the Mann–Whitney U test and categorical variables using Fisher’s exact test. Univariate associations were examined with logistic regression or Cox proportional-hazards models as appropriate. Overall and transplant-free survival were analyzed by Kaplan–Meier methods and compared with the log-rank test. Analyses were performed with IBM SPSS Statistics, version 29.0 (IBM Corp., Armonk, NY, USA) and MedCalc Statistical Software, version 20.218 (MedCalc Software Ltd., Ostend, Belgium). Multivariate modeling was not undertaken because of the limited sample size. A *p*-value < 0.05 was considered statistically significant.

Artifical intelligent (AI, ChatGPT 5.6, OpenAI, San Francisco, CA, USA) was used solely to improve the clarity and grammar of the manuscript; it was not used for statistical analysis. The scientific content, analyses, and conclusions are those of the authors, who verified the text before submission.

## 3. Results

### 3.1. Cohort Characteristics

Of 1720 patients with ILD evaluated during the study period, 34 (2.0%) were positive for anti-MDA5 antibody. A comprehensive diagnostic breakdown of the remaining 1686 MDA5-negative ILD evaluations was not extracted for this analysis and is therefore not presented as a flow-diagram; those evaluations comprised the usual spectrum of idiopathic, systemic-autoimmune-related (SARD), fibrotic, and exposure related ILDs referred to the program. Table 1 summarizes the baseline demographic characteristics of this cohort, including age, sex, ethnicity, diagnostic classification, ILD/RP-ILD frequency, transplant listing, and mortality. Key laboratory, imaging, spirometric, and hemodynamic findings are presented in Table 2. Mean age was 50 years (median 51, IQR 41–60); 19 (56%) were female. Ethnicity distribution was Caucasian 12 (35%), Black eight (24%), Asian five (15%), Hispanic four (12%), other three (9%), and unknown two (6%). Twenty-two patients (65%) were first evaluated by the advanced lung disease service in the inpatient setting and 12 (35%) as outpatients.

Thirty-two patients (94%) were diagnosed with DM or CADM; two (6%) fulfilled criteria for antisynthetase syndrome (with concurrent anti-Jo-1 or anti-EJ antibodies and relatively low MDA-5 titers). Nine patients (26%) had an additional connective-tissue disease diagnosis. One patient (3%) developed central-nervous-system B-cell lymphoma during follow-up. Median time from symptom onset to diagnosis/treatment initiation was 0.5 months (mean 5.7 months).

### 3.2. Muscle Involvement

**Figure 1:**
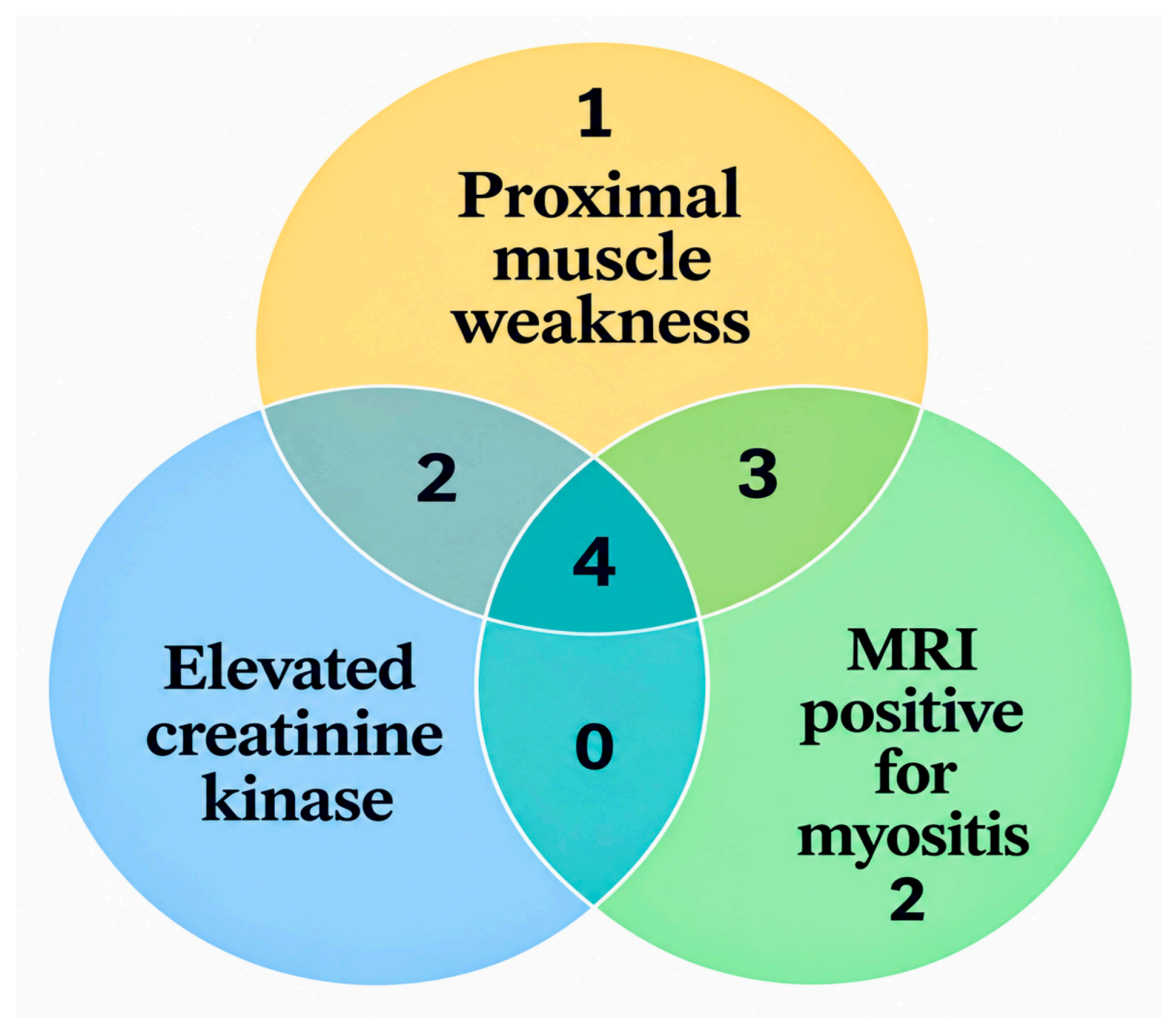
Overlap of proximal muscle weakness, elevated creatinine kinase and MRI findings consistent with myositis among patients who underwent MRI (*n* = 14). Numbers indicate patient counts in each region of the Venn diagram.

Thigh MRI was performed in 14 patients (41%); 9/14 (64%) showed findings consistent with myositis. Muscle biopsy was obtained in seven patients (21%); 5/7 (71%) confirmed myositis, including two with necrosis. The overlap of clinical, laboratory and imaging findings of muscle disease is illustrated in Figure 1.

### 3.3. Pulmonary Involvement and Biomarkers

ILD was present in 29 patients (85%). RP-ILD occurred in 13 patients (38%). One patient presented with acute-on-chronic deterioration; after multidisciplinary review, this patient was classified as RP-ILD because respiratory decline occurred over less than six weeks without an alternative explanation and despite ongoing therapy. Additional pulmonary complications included pneumomediastinum or pneumothorax (seven patients, 21%), organizing pneumonia (three, 9%), Pneumocystis jirovecii pneumonia (two), CMV pneumonitis (two), and pulmonary embolism (one).

Key spirometric, biomarker and hemodynamic data are summarized in Table 2. PFTs were available in 24 of 34 patients (71%) and showed a restrictive pattern as follows: median FVC 64% predicted (IQR 47–80), FEV1 63% (49–77), FEV1/FVC 85% (80–93), and DLCO 48% (37–63).

**Figure 2:**
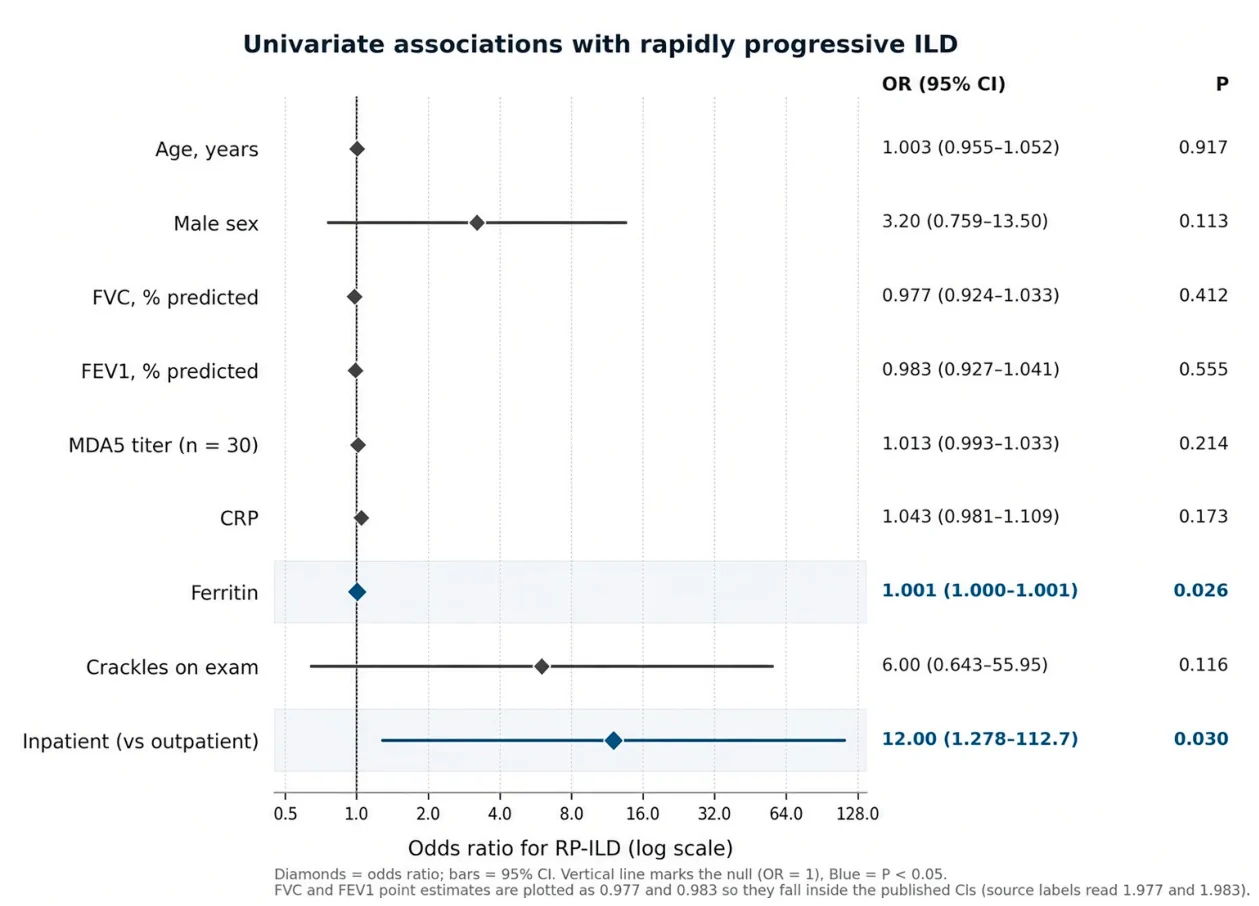
Univariate analysis of factors associated with rapidly progressive ILD (RP-ILD). Odds ratios (ORs) with 95% confidence intervals are shown.

Elevated CRP was present in 19/33 patients tested (58%); ferritin was elevated in 16/21 (76%) and CK was elevated in 8/30 (27%). Baseline CRP was significantly higher in RP-ILD than non-RP-ILD. Factors associated with development of RP-ILD on univariate analysis are shown in Figure 2; inpatient presentation and elevated ferritin reached statistical significance.

### 3.4. Pulmonary Hypertension

Right-heart catheterization was performed in 11 patients (32%). Pulmonary hypertension, defined as mPAP > 20 mmHg, was present in 7 of 11 patients (64%); most cases were pre-capillary. All patients with PH had concurrent ILD. Among patients with PH, approximate mean hemodynamic values were systolic PAP 51 mmHg, mean PAP 33 mmHg, PCWP 11 mmHg, cardiac index 2.8 L/min/m^2^ and PVR 4.1 Wood units. Formal reporting of mean ± SD versus median (IQR) was limited by the small number of catheterized patients (*n* = 11).

### 3.5. Treatment

Corticosteroids were administered to 33/34 patients (97%), including pulsed-dose intravenous methylprednisolone in a substantial proportion of the RP-ILD group. Exact cumulative corticosteroid dose was not uniformly recorded in the medical record and therefore cannot be summarized as a single mean or median value. Additional agents are summarized as *n* (%) in Table 3: mycophenolate mofetil 17 (50%), intravenous immunoglobulin 13 (38%), tofacitinib nine (26%), tacrolimus eight (24%), plasma exchange seven (21%), cyclosporine seven (21%), and rituximab six (18%). Escalation therapies were concentrated in the RP-ILD group.

**Figure 3:**
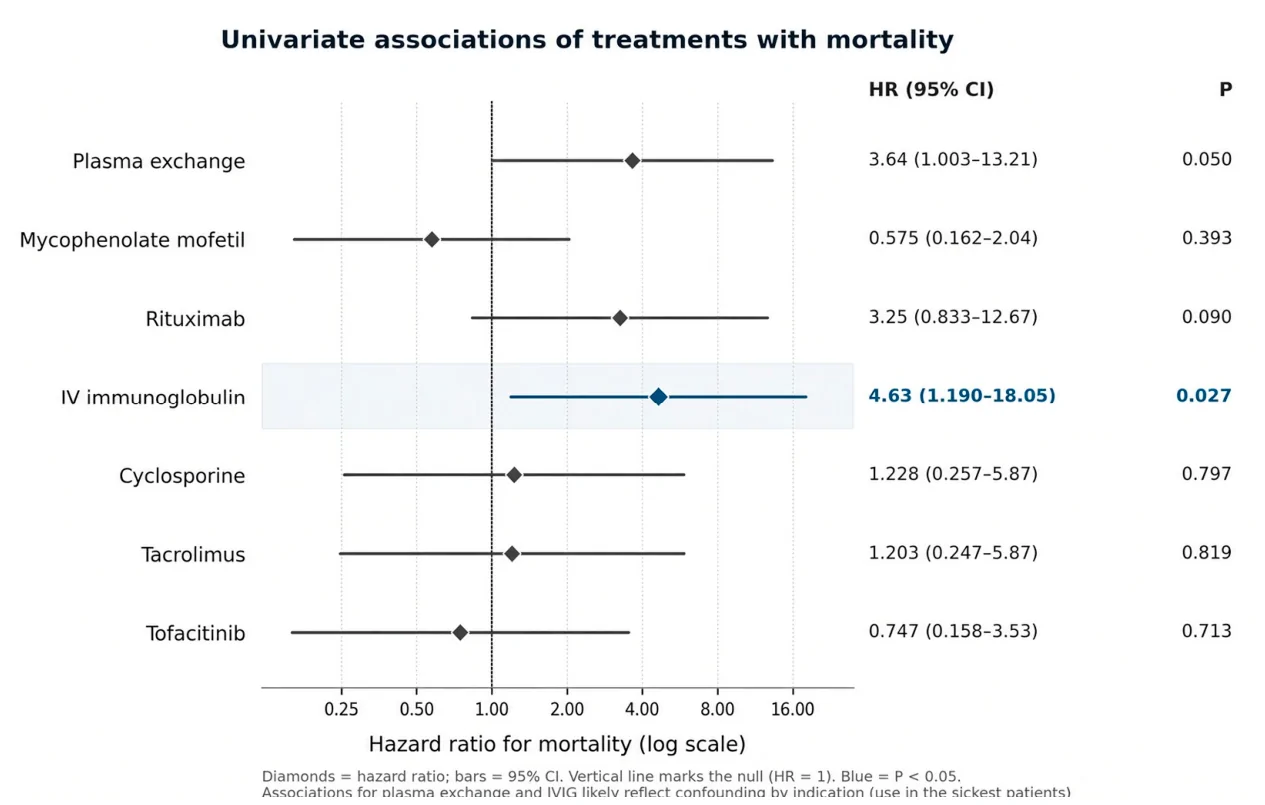
Univariate Cox regression analysis of treatments and hazard ratio (HR) for mortality. Hazard ratios with 95% confidence intervals are shown.

Univariate analysis of treatment associations with mortality is shown in Figure 3. Plasma exchange and IVIG were associated with higher hazard ratios for mortality on univariate analysis.

### 3.6. Mortality and Survival

Overall mortality was 11/34 (32%). Eleven of 13 patients with RP-ILD died (85%). There were zero deaths among the 21 patients without RP-ILD during available follow up (0%). Cause of death was respiratory failure in every case. Mean time from symptom onset to death was 7.4 months (median ≈ 7 months).

**Figure 4:**
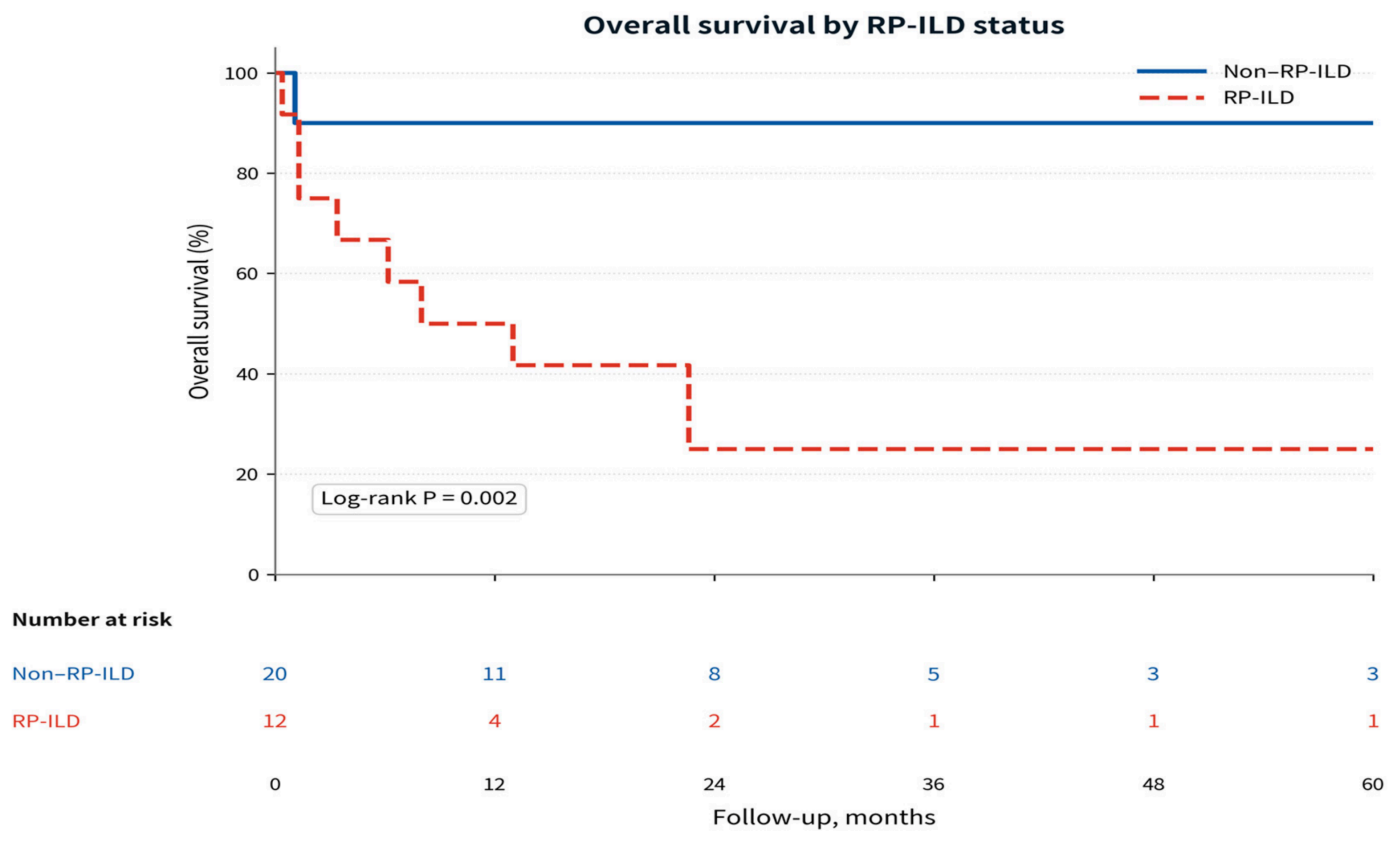
Kaplan–Meier overall survival stratified by rapidly progressive ILD (RP-ILD) status. Time zero is diagnosis/treatment initiation. Log-rank *p* = 0.002. Number at risk is shown below the x-axis.

**Figure 5:**
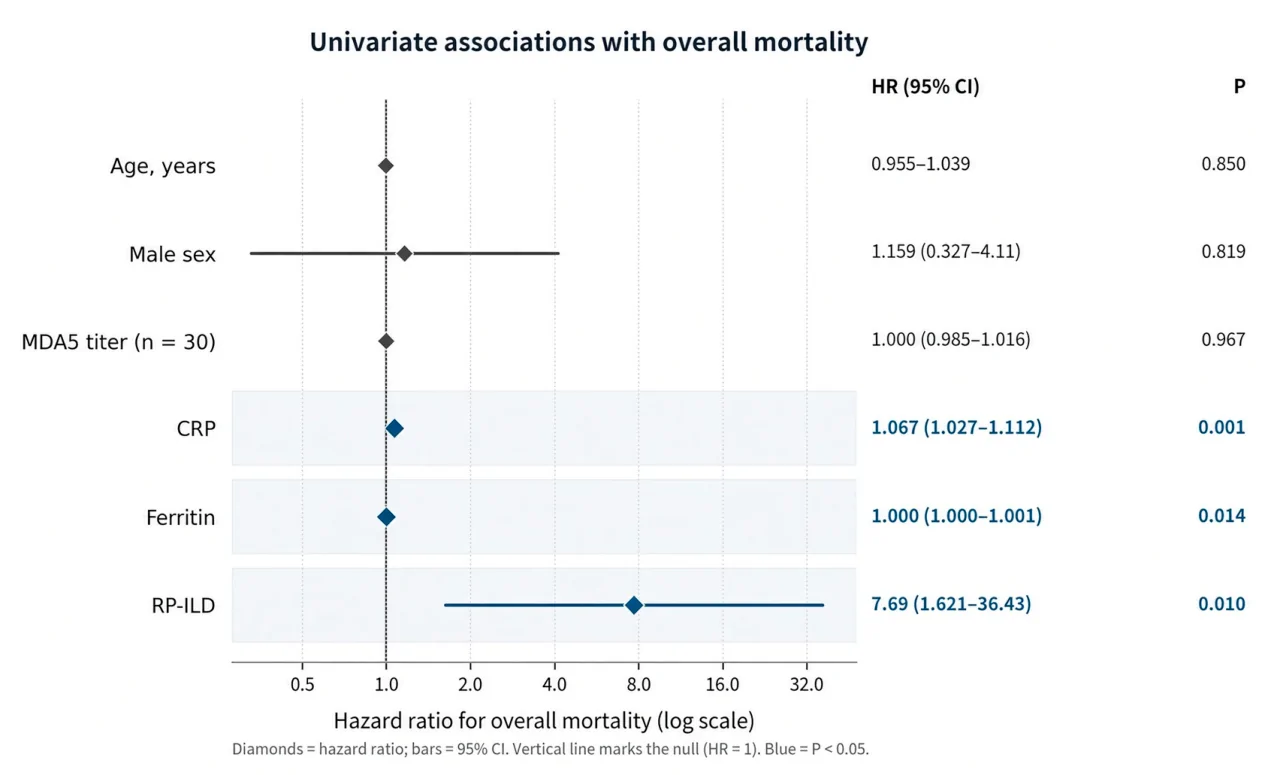
Univariate Cox regression analysis of selected variables and hazard ratio (HR) for overall mortality. Hazard ratios with 95% confidence intervals are shown.

Kaplan–Meier overall survival curves stratified by RP-ILD status are shown in Figure 4 (log-rank *p* = 0.002). One-year overall survival was substantially lower in RP-ILD. Univariate predictors of overall mortality included RP-ILD (HR 7.686, 95% CI 1.621–36.433, and *p* = 0.010) and elevated CRP (Figure 5). RP-ILD and elevated CRP were significant predictors of overall mortality on univariate analysis.

**Figure 6:**
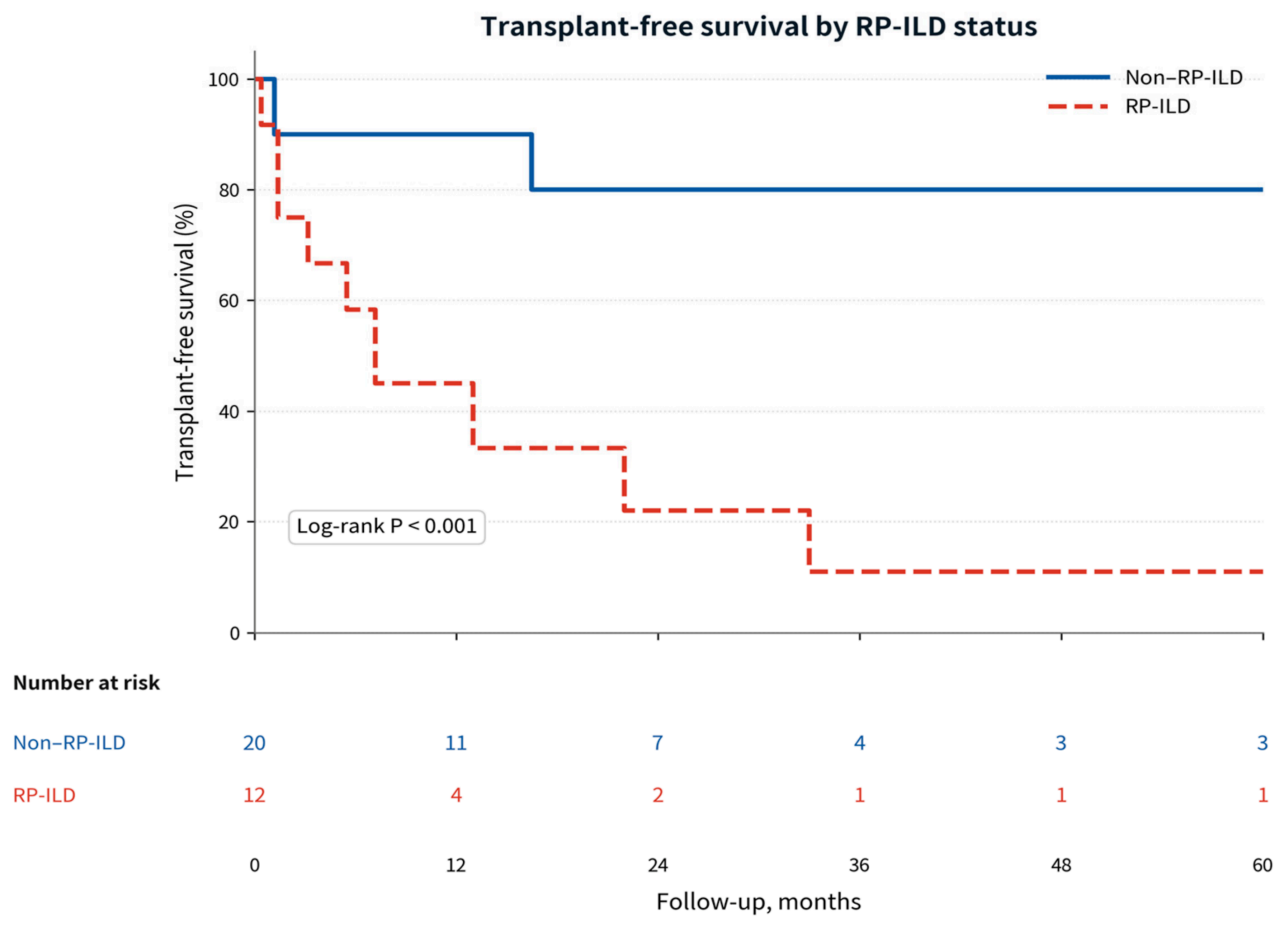
Kaplan–Meier transplant-free survival stratified by RP-ILD status. Log-rank *p* < 0.001.

**Figure 7:**
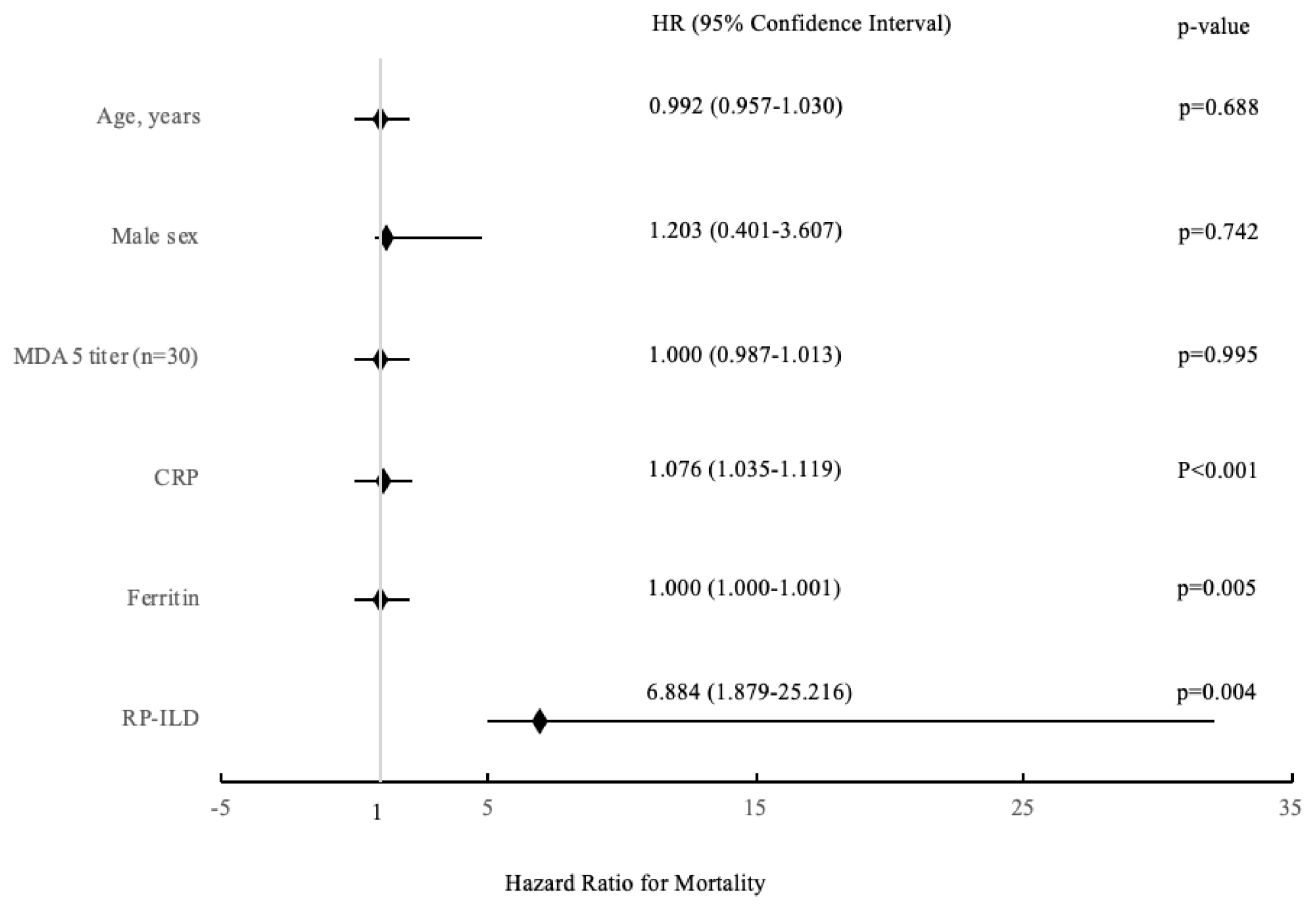
Univariate Cox regression analysis of selected variables and hazard ratio for transplant-free mortality. Elevated CRP and RP-ILD remained significant.

Transplant-free survival showed a similar pattern (Figure 6, log-rank *p* < 0.001). Univariate analysis for transplant-free mortality again highlighted CRP and RP-ILD (Figure 7). Elevated CRP and RP-ILD remained significant predictors of transplant-free mortality on univariate analysis.

### 3.7. Lung Transplantation

Six patients (18%) were listed for lung transplantation; three (9% of the cohort) underwent transplantation. Time from diagnosis to transplant was 4 months, 18 months, and 30 months in the three recipients, respectively. One recipient required prolonged veno-venous extracorporeal membrane oxygenation (ECMO) before transplant. All three transplanted patients remained alive at last follow-up (24, 31 and 49 months post-transplant). Three listed patients were subsequently delisted because of progressive critical illness, debility or frailty, underscoring the narrow therapeutic window for transplant candidacy.

## 4. Discussion

This single-center cohort from an integrated ALD/LTx Program highlights several clinically relevant observations. First, RP-ILD developed in more than one-third of anti-MDA5-positive patients and accounted for all observed deaths. Patients without RP-ILD had excellent intermediate-term survival, underscoring the prognostic importance of the rapidly progressive phenotype. These mortality findings are broadly consistent with previously published anti-MDA5 cohorts, in which short-term mortality among patients who develop RP-ILD frequently exceed 50–80% despite intensive immunosuppression, whereas patients without a rapidly progressive course generally have a more favorable trajectory [3,4,5,6,10]. Differences across series likely reflect variation in RP-ILD definitions, ethnicity, referral patterns, and treatment era, including increasing use of calcineurin inhibitors and JAK inhibitors [4,8,9,10,13]. The 32% overall mortality in the present cohort and the 85% mortality among patients with RP-ILD should therefore be interpreted as consistent with the severe end of published spectrum rather than an outlier. Because this series was assembled at a tertiary ALD/LTx Center, the findings cannot be assumed to represent all anti-MDA5-positive patients. Individuals managed exclusively in community rheumatology or general pulmonary clinics, particularly those with limited cutaneous disease and no or mild ILD, are under-represented. Referral and selection bias toward more severe pulmonary disease is therefore an important limitation and reduces generalizability of the absolute mortality estimates. Associations identified on univariate analysis, including those involving CRP, ferritin, inpatient presentation, plasma exchange, IVIG, and other escalation therapies, should be interpreted as associations rather than evidence of causality. The retrospective observational design, modest sample size, non-protocolized treatment, and confounding by indication preclude causal inference regarding the efficacy or harm of any individual agent.

Second, lung transplantation is feasible and can be lifesaving in carefully selected patients with anti-MDA5-associated ILD, including those who present with RP-ILD. In this series, six patients (18%) were listed and three (9%) underwent transplantation. Time from diagnosis to transplant was 4, 18, and 30 months. All three recipients were alive at last follow-up 24, 31, and 49 months after transplant. One required prolonged veno-venous ECMO as a bridge, indicating that transplantation remains possible after a period of extracorporeal support if systemic inflammation can be controlled and the patient can be kept a candidate. These observations are consistent with limited published experience suggesting acceptable short-term allograft outcomes and no clear pattern of recurrent anti-MDA5 ILD in the transplanted lung [11]. The listing experience also shows how narrow the window is. Three of the six listed patients were subsequently delisted because of progressive critical illness, debility, or frailty. That 50% dropout among listed patients is the practical argument for early referral, ideally at the first signal of RP-ILD, refractory hypoxemia, or failure to stabilize on combination immunosuppression, rather than after irreversible deconditioning. Referral to a center that can run advanced lung disease care, ECMO, and transplant evaluation in parallel is likely more important than any single induction regimen. These data cannot define optimal listing criteria or the best timing of ECMO; they only show that delay until the patient is too sick to remain listed is common and often fatal in this phenotype.

Third, pulmonary hypertension was common among the patients who underwent right-heart catheterization. RHC was performed in 11 of 34 patients (32%), and 7 of those 11 (64%) met current hemodynamic criteria for PH (mPAP > 20 mmHg). Most cases were precapillary. All patients with PH had concurrent ILD. Approximate mean values among those with PH were systolic PAP 51 mmHg, mPAP 33 mmHg, PAWP 11 mmHg, cardiac index 2.8 L/min/m^2^, and PVR 4.1 Wood units. These figures should not be read as a prevalence estimate for all anti-MDA5 ILD: catheterization was reserved for a minority of the cohort and was almost certainly enriched for more severe lung disease. Even with that selection bias, the frequency is high enough to justify more systematic screening for group 3 PH in this population. Anti-MDA5 ILD can progress over weeks, so a single echocardiogram at diagnosis may miss later pulmonary vascular involvement. Identification of PH associated with ILD now has therapeutic implications, because approved therapies for PH-ILD are available [12]. Whether treating PH changes survival in anti-MDA5 ILD specifically is unknown from these data. At a minimum, unexplained dyspnea out of proportion to spirometry, a falling DLCO, right-ventricular abnormalities on echocardiography, or evaluation for transplant should prompt RHC rather than attribution of all symptoms to parenchyma alone.

Fourth, anti-MDA5 antibody was detected in 34 of 1720 ILD evaluations (2.0%) at this program. The cohort was relatively young (median age 51 years; IQR 41–60), and most patients had DM or CADM, but two fulfilled criteria for an antisynthetase syndrome with concurrent antibodies and low-titer MDA5, and nine had another connective-tissue disease. Median time from symptom onset to diagnosis or treatment was only 0.5 months, which implies that many presentations were acute. That mix supports considering anti-MDA5 testing in younger adults with new or rapidly progressive ILD, particularly when the course is measured in weeks, the imaging pattern is organizing pneumonia or NSIP-like, or there are cutaneous, vasculopathic, or rheumatic features. Routine testing of every older patient with slowly fibrotic ILD is not supported by these data and would need a different study design. Assay performance also matters: positivity here was defined by the institutional commercial immunoassay and laboratory cutoff then in use, and titers were not uniformly archived. False-negative or low-titer results, and dual-antibody profiles, therefore, remain possible. The clinical payoff of earlier testing is not a change in the antibody itself but time: RP-ILD accounted for every death in this cohort, and the interval from symptoms to death among those who died was measured in months. Identifying the antibody while the patient can still tolerate combination immunosuppression or be referred for transplant is the rationale for a low threshold in the acute, younger ILD phenotype, not a claim that screening all ILD will improve population survival.

Treatment patterns reflected real-world escalation. RP-ILD patients received substantially more plasma exchange, IVIG, cyclophosphamide and tofacitinib. These associations with mortality should again be interpreted as markers of disease severity and confounding by indication rather than evidence of harm or lack of efficacy. Prospective data support early combination immunosuppression, while observational series continue to explore the role of JAK inhibitors [9,10,13].

Strengths of the study include consecutive enrolment at a transplant-capable center, detailed hemodynamic data, and complete vital-status follow-up. Limitations are inherent to its retrospective, single-center design: modest sample size, incomplete data for some variables (including continuous biomarker values, exact corticosteroid exposure, and individual antibody titers), potential referral bias toward more severe disease, variation in laboratory assays, non-protocolized treatment, and the lack of a standardized, prospectively applied RP-ILD definition. Multivariate adjustment was not feasible. Absolute mortality estimates, particularly the 85% mortality among patients with RP-ILD, cannot be directly generated to all anti-MDA5 positive patients managed outside tertiary referral centers.

## 5. Conclusions

In this single-center retrospective cohort, anti-MDA5-associated ILD remained a high-stakes diagnosis when RP-ILD supervened. Within the limits of observational data and a small sample, the findings support early recognition, intensive multi-agent immunosuppression, systematic consideration of concurrent pulmonary hypertension, and prompt evaluation for lung transplantation in selected patients. These observations should not be interpreted as proof that any specific regimen caused improved survival. Larger multicenter collaborations and prospective registries are needed to refine risk stratification and treatment algorithms.

## Figures and Tables

**Table 1:** Baseline characteristics of the anti-MDA5 cohort (*N* = 34).

Characteristic	Value
Age, years, mean/median (IQR)	50/51 (41–60)
Female sex, *n* (%)	19 (56)
Ethnicity, *n* (%)	Caucasian 12 (35), Black 8 (24), Asian 5 (15), Hispanic 4 (12), Other/Unknown 5 (15)
First evaluation setting	Inpatient 22 (65), Outpatient 12 (35)
DM or CADM, *n* (%)	32 (94)
Antisynthetase syndrome, *n* (%)	2 (6)
Concurrent autoimmune disease, *n* (%)	9 (26)
ILD present, *n* (%)	29 (85)
RP-ILD, *n* (%)	13 (38)
Listed for lung transplant, *n* (%)	6 (18)
Underwent lung transplant, *n* (%)	3 (9)
Overall mortality, *n* (%)	11 (32)
Mortality in RP-ILD, *n* (%)	11/13 (85)
Mortality in non-RP-ILD, *n* (%)	0/21 (0)

IQR = interquartile range; DM = dermatomyositis; CADM = clinically amyopathic dermatomyositis; ILD = interstitial lung disease; RP-ILD = rapidly progressive ILD.

**Table 2:** Key laboratory, diagnostic and hemodynamic findings.

Variable	Result
ANA positive	21/34 (62%)
Anti-SSA/Ro positive	19/34 (56%)
Elevated CRP	19/33 (58%)
Elevated ferritin	16/21 (76%)
Elevated CK	8/30 (27%)
MRI thighs performed/positive for myositis	14/9 (64%)
Muscle biopsy performed/positive	7/5 (71%)
RHC performed/PH present	11/7 (64%)
Median FVC % predicted (IQR)	64 (47–80)
Median DLCO % predicted (IQR)	48 (37–63)

ANA = antinuclear antibody; CRP = C-reactive protein; CK = creatine kinase; RHC = right-heart catheterisation; PH = pulmonary hypertension; FVC = forced vital capacity; DLCO = diffusing capacity of the lung for carbon monoxide.

**Table 3:** Treatments administered (*N* = 34).

Agent	*n* (%)
Corticosteroids	33 (97)
Mycophenolate mofetil	17 (50)
Intravenous immunoglobulin	13 (38)
Tofacitinib	9 (26)
Tacrolimus	8 (24)
Plasma exchange	7 (21)
Cyclosporine	7 (21)
Rituximab	6 (18)

## Data Availability

The datasets generated and analyzed during the current study contain clinical information and are not publicly available. De-identified data may be available from the corresponding author on reasonable request and with appropriate institutional approvals.
